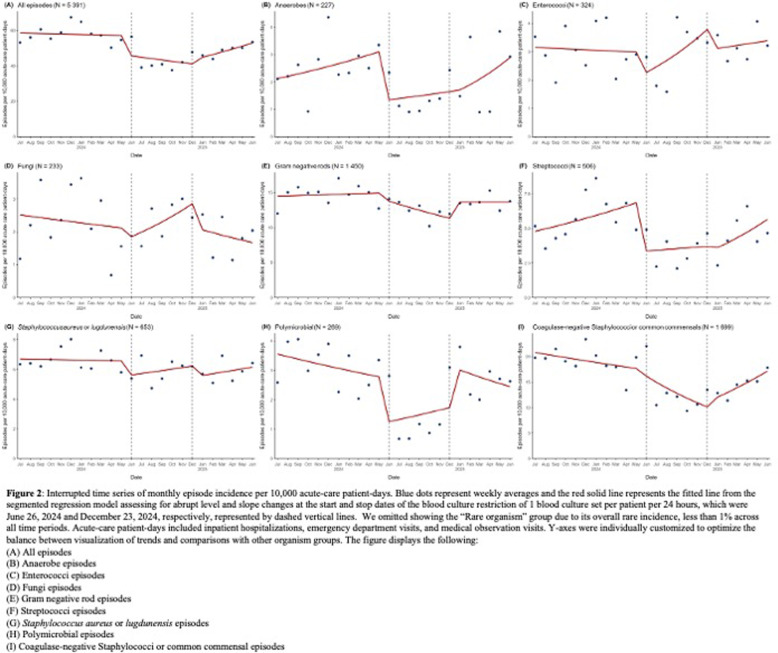# 206 Carriage of Endemic and Emerging Multidrug-Resistant Organisms (MDROs) in Ventilator-Capable Skilled Nursing Facilities (vSNFs)

**DOI:** 10.1017/ash.2026.10441

**Published:** 2026-06-23

**Authors:** Joseph Ladines-Lim, Leigh Cressman, Bailey Van, Warren Bilker, Kathleen Degnan, Michael David

**Affiliations:** 1 University of Pennsylvania; 2 University of Pennsylvania/Dept. of Biostatistics, Epidemiology and Informatics; 3 Columbia University, Fu Foundation School of Engineering and Applied Science; 4 The University of Pennsylvania

## Abstract

**Background:** During the 2024 national shortage of BD BACTEC blood culture bottles, many hospitals implemented ordering restrictions to conserve supplies. While prior studies have evaluated overall blood culture utilization and patient outcomes, less is known about how such restrictions affected the observed epidemiology of bloodstream infections (BSIs) across organism groups. Understanding these effects is important for interpreting surveillance data and anticipating downstream clinical implications during diagnostic supply disruptions. **Methods:** We conducted an interrupted time series analysis of BSI episodes at two tertiary-care hospitals from June 26, 2023 through June 25, 2025. A systemwide electronic order restriction implemented from June 26, 2024 through December 23, 2024 limited clinicians to one blood culture set per patient every 24 hours. We grouped blood culture results into episodes and categorized by organism group. Outcomes included organism group-specific episode incidence, hospital outcomes (e.g., in-hospital mortality/hospice discharge), and blood culture utilization. We used segmented regression models with Newey-West standard errors to estimate level and slope changes associated with restriction onset and withdrawal. **Results:** We identified 5,391 BSI episodes among 4,484 patients, with baseline characteristics stable across study periods (Table 1). Organism group incidence, hospital outcomes, and blood culture utilization varied by period (Table 2). During the restriction period, the relative incidence of enterococci, fungi, gram negative rods, and Staphylococcus aureus/lugdunensis increased, while anaerobes, streptococci, coagulase-negative staphylococci/common commensals, and polymicrobial infections decreased (Figure 1). Interrupted time series analyses demonstrated an overall decrease in BSI incidence during the restriction, with declines for gram negative rods, streptococci, coagulase-negative staphylococci/common commensals, and polymicrobial infections (Figure 2). We observed statistical significance only for a 51.7% decrease in streptococci incidence upon restriction onset (P=.04) and a 3.5% per month decrease in gram negative rod incidence during restriction (P=.02) (Table 3). Estimates for other organism groups were limited by small numbers of observations, resulting in imprecise effect estimates or potentially artifactual findings. Conclusions Single-set blood culture restriction during the 2024 national supply shortage was associated with changes in the observed epidemiology of BSIs, including an overall decrease in incidence and heterogeneous effects across organism groups. Although interrupted time series analyses for several groups were limited by low power, combined visual and quantitative findings suggest that restrictive diagnostic practices may differentially affect BSI detection by organism type. These findings underscore the need for cautious interpretation of surveillance data during diagnostic disruptions and highlight the importance of resilient diagnostic supply chains and evidence-based mitigation strategies.

**Figure:**
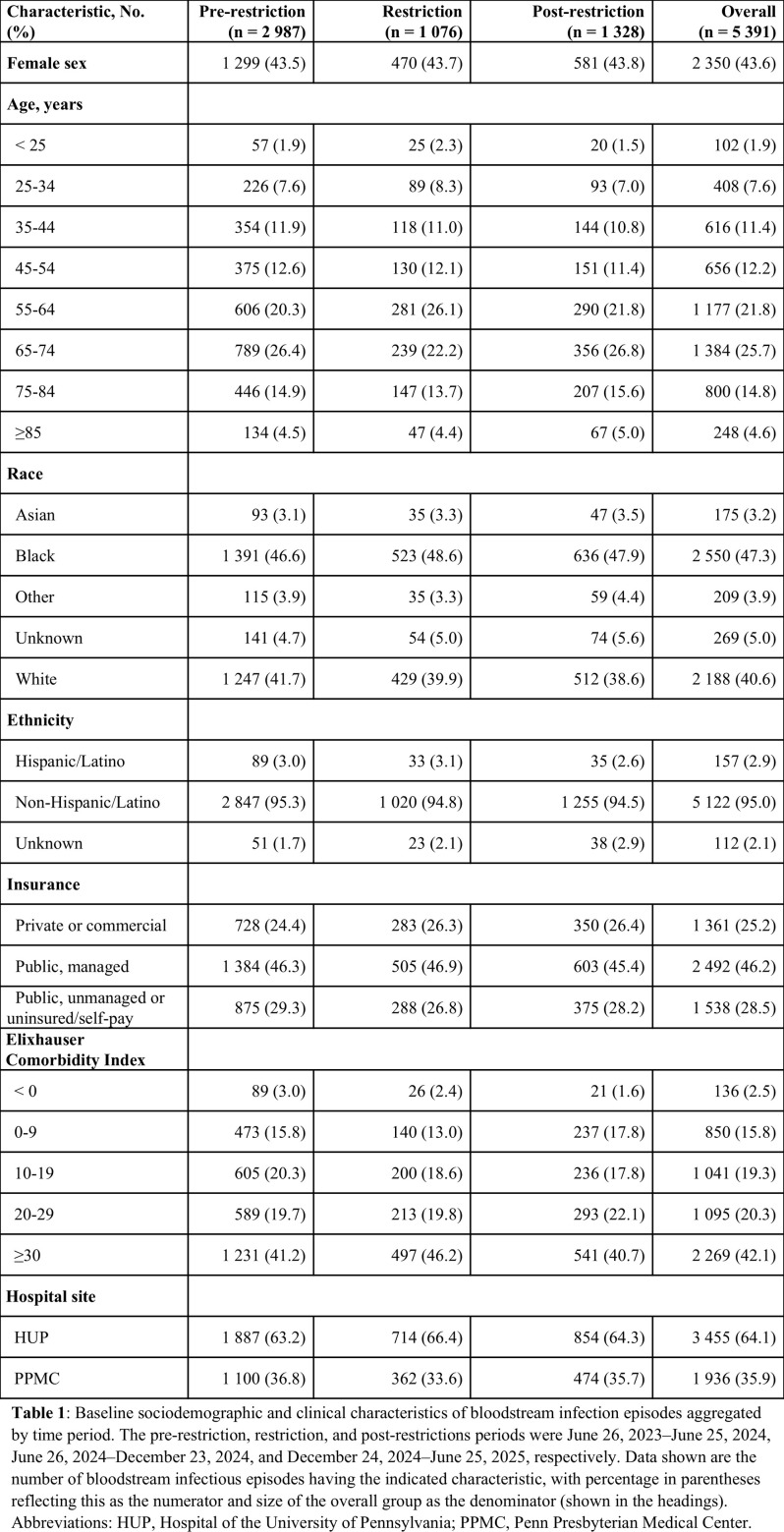

**Figure:**
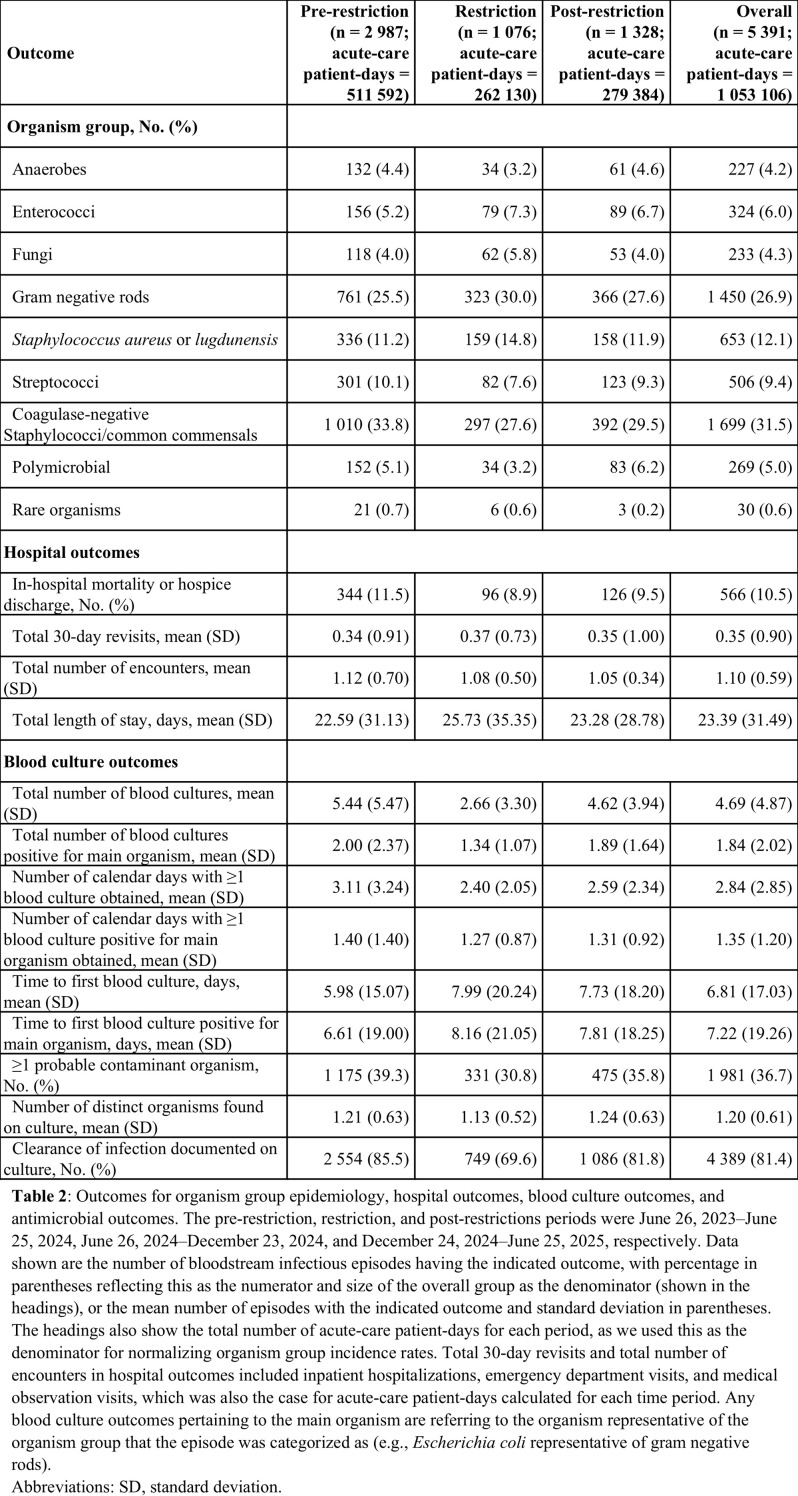

**Figure:**
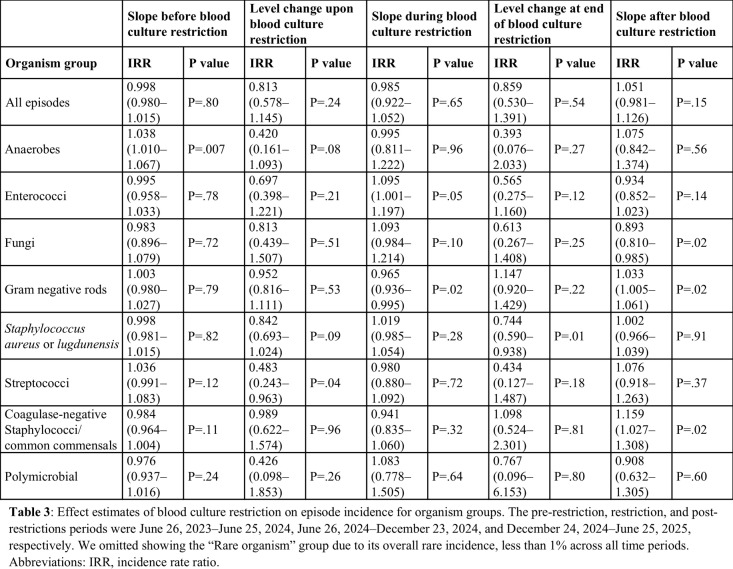

**Figure:**
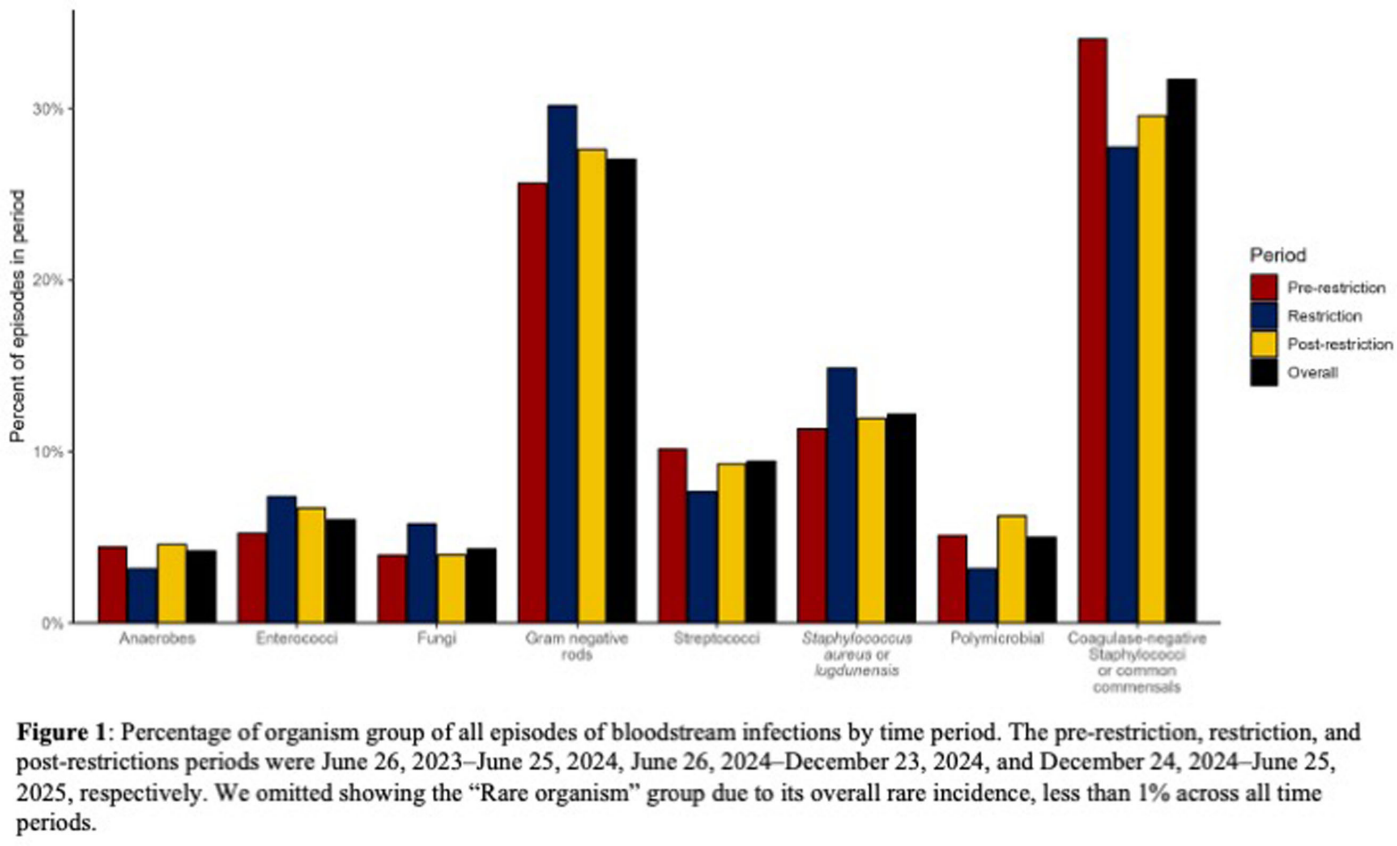

**Figure:**